# Complete mitochondrial genomes of *Trisidos kiyoni* and *Potiarca pilula*: Varied mitochondrial genome size and highly rearranged gene order in Arcidae

**DOI:** 10.1038/srep33794

**Published:** 2016-09-22

**Authors:** Shao’e Sun, Qi Li, Lingfeng Kong, Hong Yu

**Affiliations:** 1 Key Laboratory of Mariculture, Ministry of Education, Ocean University of China, Qingdao 266003, China

## Abstract

We present the complete mitochondrial genomes (mitogenomes) of *Trisidos kiyoni* and *Potiarca pilula*, both important species from the family Arcidae (Arcoida: Arcacea). Typical bivalve mtDNA features were described, such as the relatively conserved gene number (36 and 37), a high A + T content (62.73% and 61.16%), the preference for A + T-rich codons, and the evidence of non-optimal codon usage. The mitogenomes of Arcidae species are exceptional for their extraordinarily large and variable sizes and substantial gene rearrangements. The mitogenome of *T. kiyoni* (19,614 bp) and *P. pilula* (28,470 bp) are the two smallest Arcidae mitogenomes. The compact mitogenomes are weakly associated with gene number and primarily reflect shrinkage of the non-coding regions. The varied size in Arcidae mitogenomes reflect a dynamic history of expansion. A significant positive correlation is observed between mitogenome size and the combined length of *cox1-3*, the lengths of *Cytb*, and the combined length of rRNAs (*rrnS* and *rrnL*) (*P* < 0.001). Both protein coding genes (PCGs) and tRNA rearrangements is observed in *P. pilula* and *T. kiyoni* mitogenomes. This analysis imply that the complicated gene rearrangement in mitochondrial genome could be considered as one of key characters in inferring higher-level phylogenetic relationship of Arcidae.

Mitochondrial DNA (mtDNA) is the only extranuclear genome in animal cytoplasm^1^. Most metazoan mitochondrial genomes (mitogenomes) are covalently closed circular molecules which range from 14 to 42 kb in length^2^, but see some Arcidae bivalves^3^,^4^. The mitogenome generally encodes for 37 genes: 13 protein-coding genes (PCGs), 2 ribosomal RNA genes (rRNAs), and 22 transfer RNA genes (tRNAs)^1^,^5^,^6^. In general, there are few intergenic nucleotides except for a single large non-coding region generally thought to contain elements that control the initiation of replication and transcription of the mitogenome^7^. Owing to the abundance of mitochondria in cells, maternal inheritance, absence of introns, and higher evolutionary rates, the mitogenomes of Metazoa are good model systems for comparative and evolutionary genomic studies^8^. Several features, such as genome size, gene arrangement, gene number and structure, can be easily and systematically investigated in the small mitogenome.

Mollusca is a megadiverse phylum originated in the PreCambrian/Cambrian border, according to the fossil record^9^, during which organisms conquered a great variety of habitats, food habits and evolved a variety of body sizes^10^. Bivalve mollusks present one of the most variable mitogenomes described among metazoans with low conservation of genome size, gene arrangement, strand assignment, gene duplications and losses, nucleotide composition, and more^6^,^8^. The mitogenomes of relatively large size (greater than 20 kb) have been found in bivalves such as the deep sea scallop *Placopecten magellanicus* (up to 40,725 bp)^11^, the Zhikong scallop *Chlamys farreri* (21,695 bp)^12^, the Manila clam *Venerupis philippinarum* (22,676 bp in female type, 21,441 bp in male type)^13^, and the Arcidae species, *i.e. Scapharca broughtonii* (46,985 bp)^3^, *Scapharca kagoshimensis* (46,713 bp)^4^, *Tegillarca granosa* (31,589 bp)^14^ and *Anadara vellicata* (34,147 bp)^15^. Size variation in bivalves mitogenomes is usually due to the different length of the non-coding regions. A peculiar way of mitochondrial inheritance, doubly uniparental inheritance (DUI), has been found in *V. philippinarum*, which may also influence the genome size in male and female mitochondrial DNA^13^,^16^,^17^,^18^.

Gene arrangements appear to be dramatically variable in the major groups of bivalves, even with differences in the same family or genus^8^,^19^,^20^. Pectinidae species seem to be a good example to prove this. Comparing gene orders of *C. farreri, Argopecten irradians, Mimachlamys nobilis* and *P. magellanicus*, even after excluding the tRNA genes from the comparison, the four mitogenomes still show no identical gene arrangement^21^. In bivalves, it is also common that species belonging to the same genus have different gene orders. For example, in *Crassostrea* congeners, *C. virginica* and the six Asian *Crassostrea*, only protein-coding gene is arranged in an identical order, but tRNAs are extensively rearranged^22^. Gene order of the mitogenome can be used to investigate evolution of organisms and of their genomes by providing (1) characters that can be used in phylogenetic analysis of ancient lineages and (2) information that can be used to develop models for the mechanisms involved in gene rearrangement, replication, and regulation^23^.

The family Arcidae belongs to the superfamily Arcacea in the order Arcoida. The species of Arcidae are globally distributed, predominantly in the tropical shallow waters and warm temperate seas, containing approximately 260 species and 31 genera^24^. Arcidae is subdivided in two subfamilies, Anadarinae and Arcinae, based on the strength of the byssus^25^. Arcinae contains some of the best-known and most widely distributed genera, like Arca (Linnaeus, 1758) and Barbatia (Gray, 1842). In Anadarinae, several species have significant economic value. For example, *Tegillarca granosa* is cultivated on wide mudflats in South-East Asia (China, Taiwan, Korea, Malysia and Thailand) and has been consumed by humans for centuries^26^; *Scapharca* species are harvested in Japan and China. At present, four complete mitogenomes, were available from this family, *i.e.*, *S. brough*tonii (GenBank: AB729113), *S. kagoshimensis* (GenBank: KF750628), *T. granosa* (GenBank: KJ607173) and *A. vellicata* (GenBank: KP954700). Obvious differences in mitogenome organization of the Arcidae species were observed: (1) the sizes of three mitogenomes are distinct from each other, *i.e.* 46,985 bp for *S. kagoshimensis*, 46,713 bp for *S. kagoshimensis*, 31,589 bp for *T. granosa* and 34,147 bp for *A. vellicata*; (2) the genomes show distinct gene arrangement patterns, namely unique rearrangements involving the tRNA genes. These variation of genomic organization provide a good system to understand the evolutionary history of the mitochondrial genomes.

In this work, we present the complete mitochondrial genomes of *T. kiyoni* and *P. pilula*, both important species from the family Arcidae (Arcoida: Arcacea). The characterization of the evolution and structural organization of *T. kiyoni* and *P. pilula* mitochondrial genomes were analyzed and compared with other Arcidae mitogenomes. We discussed our findings with particular reference to the variations in genome size and gene arrangement in the family Arcidae. We reconstructed the phylogenetic relationships of six Arcidae species based on twelve protein-coding genes, which allows for the understanding of ancestral organization of the Arcidae mitogenomes.

## Results and Discussion

### Genome organization, structure and composition

**Figure 1:**
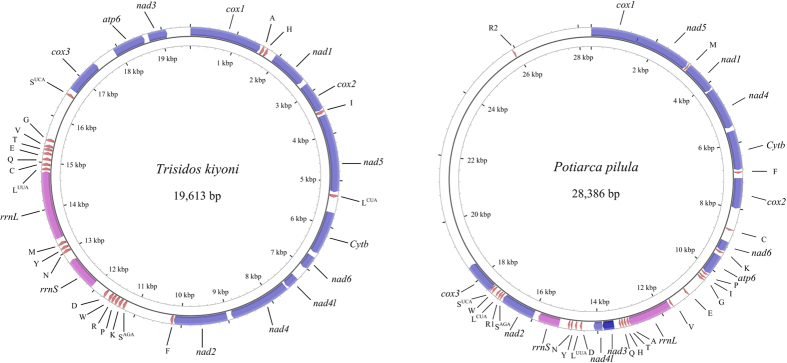
The organization of the mitochondrial genome of *Trisidos kiyoni* and *Potiarca pilula*. Genes for proteins and rRNA (*rrnS* and *rrnL*) are listed under abbreviations. Transfer-RNAs are represented by their one-letter amino acid code.

The complete mtDNA sequences of *T. kiyoni* and *P. pilula* are 19,614 bp and 28,470 bp in size, respectively, and their structural organization are depicted in Fig. 1, Tables 1 and 2. The mitochondrial genome sequence of *T. kiyoni* is the smallest in all Arcidae mitochondrial genomes available in the GenBank. The *T. kiyoni* mitogenome contains 12 PCGs, 22 tRNA genes, 2 rRNA genes and non-coding regions. Unlike *T. kiyoni*, *P. pilula* mitogenome contains 23 tRNA genes (the standard 22 tRNAs and an extra *trnR*). No *atp8* coding sequence was detected in both *T. kiyoni* and *P. pilula* mitogenomes. All the genes are transcribed from the (+) strand of the molecules. The two mitogenomes exhibit different gene arrangements for both tRNA and protein-coding genes with other Arcidae mitogenomes^3^,^4^,^14^,^15^.

In the *T. kiyoni* mitogenome, there are 36 non-coding regions with a total of 5,369 bp long varying from 2 bp to 1009 bp. The longest non-coding region is situated between *trnF* and *trnS*^*AGA*^. The *P. pilula* mitogenome contains 34 non-coding regions with a total of 13,642 bp with various lengths of 0–7,408 bp. The two long non-coding regions are located between *cox3* and *trnR2* (7,408 bp), *trnR2* and *cox1* (2,435 bp), respectively. In *P. pilula*, the overlaps occur two times and involve a total of 45 bp, which located between *cox1* and *nad5* (31 bp), *trnS2* and *cox3* (14 bp). There is no overlapping gene in the mitochondrial genome of *T. kiyoni*. The overlap between *cox1* and *nad5* was also observed in the mitogenome of *S. broughtonii* and *A. vellicata*^3^,^15^.

The A + T content, AT-skew, and GC-skew are three parameters, which were usually used in the investigation of the nucleotide-compositional behavior of mitochondrial genomes^27^,^28^. The nucleotide compositions of the complete mtDNA sequence for both of the Arcidae species are biased toward A and T (Table 3). The A + T content is 62.73% in *T. kiyoni* and 61.16% in *P. pilula*. The non-coding region (NCR) show the highest A + T content (67.15% and 63.09%, respectively). In order to evaluate the base bias in the mitogenomes, we measured skewness in different gene regions of *T. kiyoni* and *P. pilula* mitochondrial genomes, and found the values of the AT-skew were mostly negative, as well as values of the GC-skew were all positive (Table 3).

### Protein-coding genes and ribosomal RNA genes

The entire length of the PCGs of *T. kiyoni* was 10,545 bp, while that of *P. pilula* was 11,151 bp. The overall A + T content of the 12 PCGs was 61.63% in the *T. kiyoni* mitogenome, ranging from 59.91% (*nad3*) to 64.72% (*atp6*). In *P. pilula* mitogenome, the A + T content of the 12 PCGs was 60.36%, ranging from 57.01% (*co2*) to 63.58% (*nad2*).

In the mitochondrial genomes of *T. kiyoni* and *P. pilula*, all of the 12 PCGs have complete start codons e.g. ATG and ATA (Table 1). In the *T. kiyoni* mitochondrial genome, eight and four PCGs started with ATG and ATA, respectively, while in the *P. pilula* mitochondrial genome, five and seven PCGs started with ATG and ATA, respectively. All the 12 PCGs genes have complete stop codons, e.g. TAA, TAG.

A total of 3515 and 3717 amino acids are encoded in *T. kiyoni* and *P. pilula* mitogenomes, respectively. The codon usage of *T. kiyoni* and *P. pilula* mitochondrial genomes (Table 4) are similar to that of other Arcidae species. All codons are used in both of the mitogenomes but with different frequencies. Amino acids coded by A + T-rich codon families (e.g. Phe, Tyr and Lys) are more frequent than amino acids coded by G + C-rich codon families (e.g. Pro and Arg). The ratio G + C/A + T-rich codons was 0.43 in *T. kiyoni* mitogenome, which is lower than that of *P. pilula* mitogenome. In both of the mitogenomes, G-ending codons are most abundent in NNY codon families and T-ending codons are most abundent in NNR and NNN codon families, and consequently, the (+) strands are T and G-rich, outlines another bias of Arcidae codon usage. There are 1.7 times more G than C and 2.7 times more T than A in the neutral sites of *T. kiyoni*. In the case of *P. pilula*, 3.4 times more G than C and 1.6 times more T than A were found at at the strand neutral sites. Codon usage bias was also observed in the vertebrate mitochondrial genomes, in which the two strands are exposed to different mutational pressures during replication, leading to an increased frequency of A and C in the (+) strand (or L-strand, in case of vertebrates)^27^,^29^,^30^. However, the Arcidae mtDNA showed the accumulation of T and G in the (+) strand, suggesting that a reversal of strand asymmetry have occurred in the members of these taxa.

**Figure 2:**
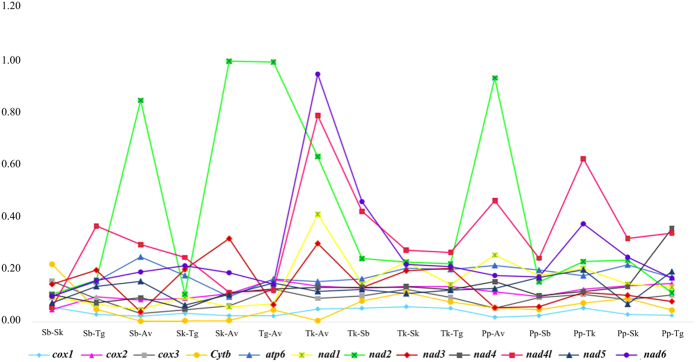
The ratios of nonsynonymous and synonymous substitution (Ka/Ks) estimated in all twelve protein coding genes of six Arcidae species.

The nonsynonymous (Ka) and synonymous (Ks) substitution rates reflect the evolutionary dynamics of protein-coding sequences across closely related species^31^,^32^. In order to detect the influence of selection pressure in Arcidae mitochondrial genomes, the number of Ka and Ks, and the ratio of Ka/Ks, were calculated for all pairwise comparisons among the six Arcidae (Fig. 2, Supplementary Table 1). The ratios of Ka/Ks between the 12 PCGs were all less than 1, indicating the existence of purifying (negative) selection in these species. Overall, the NADH dehydrogenase complex genes harbor more nonsynonymous substitutions than the cytochrome *c* oxidase subunit (*cox1*-*cox3*) genes and cytochrome *b*. This tendency was consistent with the hypothesis that the genes coding for the three subunits of the cytochrome *c* oxidase and cytochrome *b* had a higher degree of conservation than the NADH dehydrogenase genes^33^. Interestingly, *nad2* showed an exceptionally high relative proportion of nonsynonymous changes and higher Ka/Ks ratio compared to the other mitochondrial coding genes. This pattern was also observed in vertebrate mitochondrial genomes, e.g. fishes, and may be associated with the distance from the origin of replication^34^. *nad2* are found immediately upstream the major non-coding region (MNR), suggesting a minimum distance from the origin of replication. Thus, during replication, it will exposed as single-stranded for longer time compared to the other genes, rendering it more likely to accumulate mutations in the highly mutagenic environment of the mitochondrion^34^,^35^. Although all ratios less than 1 is consistent with purifying selection, the Ka/Ks ratio close to 1 is unusual for mt genes, positive selection cannot be ruled out entirely in *nad2* gene.

Identification of the *rrnL* genes in *T. kiyoni* and *P. pilula* were accomplished by comparison with other Arcidae *rrnL* genes. A conserved 23 bp-long sequence ‘AGGAGTACGGGAACGTGCCTCCT’ was used to identify the 3′ end of *rrnS* gene in *T. kiyoni* and *P. pilula*. This motif was conserved and also reported as the basis to infer the 3′ end of *rrnS* in other Arcidae mitogenomes^15^. The length of *rrnL* is 1,479 bp, and the *rrnS* is 710 bp in *T. kiyoni*. They are the largest rRNA genes yet reported in the family Arcidae. In *P. pilula*, the *rrnL* and *rrnS* are 1,344 bp and 673 bp in length, respectively (Table 1).

### Transfer RNA genes and anticodons

The complete set of 22 tRNA genes typical of metazoan mitogenomes were present in *T. kiyoni*: two tRNAs for each of serine and leucine, and one tRNA for each of the other 18 amino acids. The *P. pilula* mitogenome contained 23 tRNAs, including the standard 22 tRNAs set and an extra *trnR*. All tRNAs were interspersed between the rRNAs and the protein-coding genes with the ranges from 64 bp (*trnS*^*UCA*^) to 74 bp (*trnI*) in *T. kiyoni* and ranged from 64 bp (*trnR2*) to 73 bp (*trnW*) in *P. pilula*. The predicted secondary structures of tRNAs in two Arcidae mitogenomes were shown in Supplementary Figs 1 and 2. Most of them can fold into canonical clover-leaf secondary structures except *trnE* and *trnS*^*AGA*^ in *T. kiyoni, trnR*^*CGA*^ and *trnS*^*AGA*^ in *P. pilula*, whose paired “DHU” arm were missing, simplifing down to a loop. A modified DHU-arms of *trnE* in *T. kiyoni* is unique among molluscs. The modified DHU-arms of *trnR*^*CGA*^ is present in only few mitochondrial genomes^36^,^37^. However, missing of the “DHU” arm in the secondary structure of the *trnS* gene-*trnS*^*UCN*^ and *trnS*^*AGN*^ is common for molluscs^37^,^38^,^39^,^40^. To work in a similar way as usual tRNAs, these aberrant tRNA genes may require coevolved interacting factors or post-transcriptional RNA editing^41^,^42^,^43^.

In vertebrate mtDNAs, the most used codon in a degenerate codon family perfectly matches the anticodon of the corresponding tRNA, which is called codon-anticodon adaptation (also known as optimal codon usage)^44^. Different from the vertebrate mitochondrial genomes, non-optimal codon usage was the characteristic of Arcidae mtDNAs, and presumably other bivalves, where the most used codon does not perfectly match the corresponding tRNA anticodon in the 22 degenerate codon families (Table 4). This codon usage bias may disrupted by the A + T mutation pressure of the mitogenomes. In addition, the mitogenomes of *T. kiyoni* and *P. pilula* shared the same tRNA anticodons with vertebrate (Table 4) suggesting the anticodon evolution in metazoan mitochondrial genomes could be under the same operational forces^45^. This result is not consistent with the hypothesis that the biased codon usage drives the evolution of tRNA anticodons in the vertebrate mitogenome^30^.

### Non-coding regions

36 non-coding regions, totaling 5,369 bp, were interspersed throughout the *T. kiyoni* mitogenome; the corresponding values were 34 and 13,642 bp for *P. pilula*. The non-coding sequences are generally rare and characterized by fewer nucleotides in *T. kiyoni*. However, it is important to notice the presence of a relatively large non-coding region in the *P. pilula* mtDNA. The A + T content of non-coding regions in *T. kiyoni* and *P. pilula* are 67.15% and 63.09%, respectively, with both negative AT-skew (−0.25 and −0.08) and positive GC-skew (0.59 and 0.46).

The largest non-coding region with increased A + T composition is considered as the control region as it usually contain the signals for replication and transcription^2^. It shows a higher size variation than the other regions of the mitogenome due to both length variation with tandem repeat units (TRs) and differences in their copy numbers^46^. In the *T. kiyoni* mitogenome, one 770-bp tandem repeat (10,333–11,102), comprising three nearly identical motifs was found in the largest non-coding region (1,009 bp) between *trnF* and *trnS*^*UCU*^. Most of the non-coding sequences (9,843 bp) were observed within one segment in *P. pilula* mitogenome, within this segment all of the sequence, except *trnR*^*CGA*^, were predicted to be non-coding DNA. The large concentrated non-coding region of *P. pilula*, contained two distinct tandem repeat units (19,804–20,936 and 25,978–26,585), which were 1,133 bp and 668 bp in length, respectively. The first repeat family contained four nearly identical motifs. The second one had a three identical copies and a third copy with a 40% length of a 180-base sequence. Tandem repeat units within non-coding regions seem a common feature in Arcidae mitogenomes, despite different length and copy number in the repeat units^3^,^4^,^14^. The tandem repeat region was also found in other molluscs^38^,^47^,^48^,^49^,^50^. The occurrence of tandem repeats could be explained by mtDNA replication through slipped-strand mispairing^51^. Stem-loop structures were detected in the tandem repeat region of *T. kiyoni* and *P. pilula* (Supplementary Fig. 3). It has been demonstrated that the potential stem-loop structures in repeated unita and its flanking part may cause an increase in slipped-strand mispairing frequency^51^,^52^.

### Varied genome size of Arcidae species

Arcidae mitochondrial genomes are exceptional for their extraordinarily large and highly variable sizes. They house by far the largest known metazoan mitochondrial genomes, with sizes ranging from 19.6 to 47 kb among the four genomes sequenced to date (https://www.ncbi.nlm.nih.gov/). Arcidae mitogenomes possess an average length of 34.5 kb, whereas *T. kiyoni* and *P. pilula* showed the length of 19,614 bp and 27,895 bp, respectively, which was the smallest characterized mitochondrial genomes in Arcidae. The smallest genome-size is weakly associated with gene number and primarily reflect shrinkage of the non-coding regions. Genomic coverage by mitochondrial non-coding regions are only 27.37% for *T. kiyoni* and 40.84% for *P. pilula*, which were much lower than that of other Arcidae.

**Figure 3:**
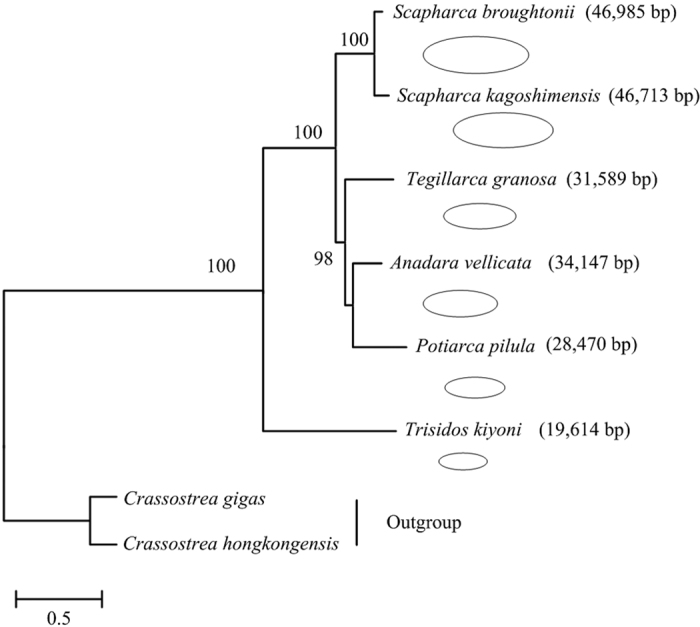
Evolution of mitochondrial genome size in Arcidae. They were shown proportional to oval size. Phylogenetic relationships derived from maximum likelihood (ML) analyses was constructed with twelve protein-coding genes (except *atp8* gene). Numbers in the nodes correspond to ML bootstrap proportions. Dashes indicate support values below 50%.

The early diverging phylogenetic positions of *T. kiyoni* within the Arcidae is such that this species provides an important insight into the historical information of Arcidae mitochondrial genomes (Fig. 3). Although it is difficult to reconstruct with the limited genomes, however, the diversity of mitogenome size among the species appears to reflect a dynamic history of expansion. The common ancestor of Aridae, like *T. kiyoni*, might have possessed a relatively compact mitochondrial genome, with a series of independent expansions leading to the large genomes in other species. However, the sources underlying major expansions in mitochondrial genome size are unknown.

**Figure 4:**
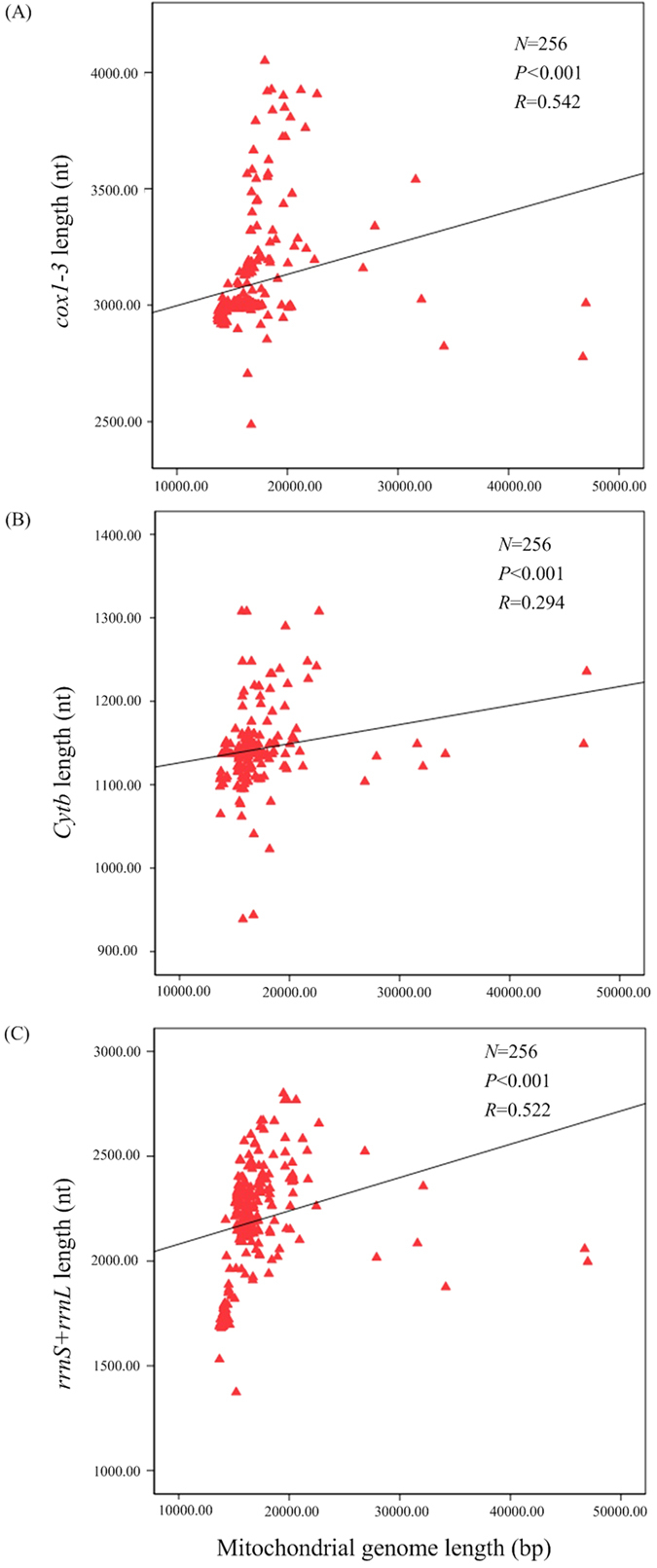
Covariation of complete mitochondrial genome size with gene lengths. Gene lengths of *cox1-3* (**A**), *Cytb* (**B**), and rRNA (*rrnS* + *rrnL*) (**C**) of different species are plotted against the corresponding complete mitochondrial genome size. The lines have been fitted by linear regressions. For species included in the analysis see supporting table in the Supplementary Table 2.

Already in 1991 it was reported that the length of mitochondrial rRNAs is correlated with the size of their corresponding organellar genomes in seven species^53^,^54^. Highly significant positive correlations were detected between mitochondrial genome size and the combined length of *cox1-3*, the length of *Cytb*, and the combined length of rRNAs (*rrnS* and *rrnL*) in 278 eukaryotes and 11 a-proteobacteria. The six mitochondrial genes are essential for oxidative phosphorylation, which in most species are refractory to nuclear transfer^54^. We presented here an analysis of this observation for 256 molluscs using six mitochondrial genes (*cox1-3*, *Cytb*, *rrnS* and *rrnL*). They have rarely been transferred to nucleus and are therefore well suited to test the hypothesis on the evolution of gene length in mitochondria^54^. A significant positive correlations are observed between the size of their mitochondrial genome and the combined length of *cox1-3*, the lengths of *Cytb*, and the combined length of rRNAs (*rrnS* and *rrnL*), which is consistent with former reports (Fig. 4, Supplementary Table 2). In many mitochondrial genomes, redox reactions produce oxygen free radicals during respiration, making a higher mutation rate than their corresponding nuclear DNA^55^. Müller’s ratchet states that these deleterious mutations can accumulate and lead to a mutational meltdown if recombination (either within or between organelles) never occurs^56^. Müller’s ratchet explains that the shorter genes may accumulate slightly deleterious mutations slower^54^. Further, the replication advantage hypothesis states that a smaller mitochondrial genome would be selected in intracellular competition due to its faster duplication rate. And this selection for smaller genomes generally contributes to the elimination of redundant cytoplasmic genes, by selecting for deletions in organelles^56^. In turn, a shorter gene might give its carrier a replication advantage during intracellular competition^54^. Thus, both of the two hypotheses predict a positive covariance between genome size and gene length. It is also supported by the shape of the gene length-genome size relationship investigated here, which is strongly asymptotic for all gene.

### Gene rearrangement as a novel structure

The mitogenomes of Bivalvia show substantially gene rearrangements, having no obvious common pattern in the arrangement of PCGs and rRNA^9^. Species sequenced in Bivalvia belong to three five subclasses: Palaeoheterodonta, Heterodonta, Pteriomorphia, Anomalodesmata and Protobranchia. Gene arrangement in Unionoida (Palaeoheterodonta) is relatively conserved, except for the translocation of several tRNAs, and protein-coding genes *nad2* and *nad3*^20^. In addition, only one mitochondrial genome is available in both Anomalodesmata (*Laternula elliptica*, KF534717) and Protobranchia (*Solemya velum*, JQ728447), and more sequences are need for further analyze. The mitochondrial gene order of the remaining bivalves is frequently rearranged, but see oysters^22^, especially for the family Pectinidae^20^,^57^.

**Figure 5:**
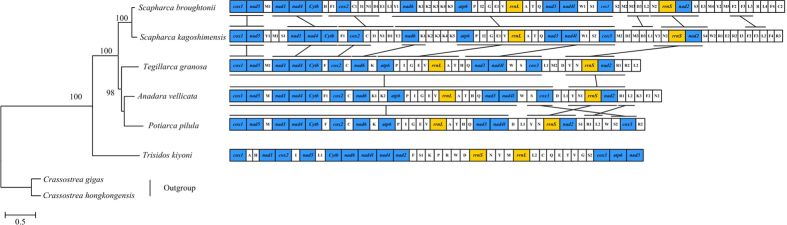
Linear representation of the mitochondrial gene arrangement in six Arcidae bivalves. *Cox1* has been designated the start point for all genomes. All genes are transcribed from left-to-right. Genes for proteins and rRNA (*rrnS* and *rrnL*) are listed under abbreviations. Transfer-RNAs are represented by their one-letter amino acid code. The non-coding regions are not presented and gene segments are not drawn to scale. The bars indicate identical gene blocks. Phylogenetic relationships derived from maximum likelihood (ML) analyses was constructed with twelve protein-coding genes (except *atp8* gene). Numbers in the nodes correspond to ML bootstrap proportions. Dashes indicate support values below 50%.

Although variability in gene arrangement is high, there are some conserved gene blocks within these groups. Arcidae seems to represent another example, as *T. kiyoni* mitogenome shared no gene block with any other five Arcidae species, despite being number of the same family, suggesting that gene rearrangements occurred dramatically among lineages in this family (Fig. 5). There were both PCGs and tRNA rearrangement in *P. pilula* mitogenome. In terms of gene arrangement, it is clear that *P. pilula* is more similar to *A. vellicata* and *T. granosa* than to *S. broughtonii* and *S. kagoshimensis*. They share three identical gene blocks: two large blocks *cox1-nad5-trnM-nad1-nad4-Cytb-trnF-cox-trnC-nad6-trnK* and *atp6-trnP-trnI-trnG-trnE-trnV -rrnL-trnA-trnT-trnH-trnQ-nad3-nad4l*, and one small block *trnY-trnN-rrnS-nad2*. If the tRNA genes are not considered, the gene order in *P. pilula* is nearly identical to that of *A. vellicata*, *T. granosa*, *S. broughtonii* and *S. kagoshimensis*, except for the translocation of gene *cox3*. For generation of the gene arrangement of mt DNA during evolution, a model involving slipped-strand mispairing of two homologous regions and random gene loss was proposed^58^,^59^. We suspect that the asymmetric gene replication and transcription accelerate this phenomenon in the evolutionary process. This hypothesis is supported by the fact that all of the mt genes of marine bivalves are encoded on same strand and show tremendous rearrangements^60^. On the other hand, the family Unionidae with dual-strand coding have relatively fewer rearrangements of gene order^20^.

Gene arrangement comparisons may be a useful tool for phylogenetic studies. This is based on the hypothesis that gene arrangements are likely to be shared only as a result of common ancestry since it is highly unlikely that the same gene order would arise independently in separate lineages^20^,^58^. We present a schematic representation of mitochondrial gene arrangements in Arcidae species on the phylogenetic trees inferred from the nucleotide dataset of 12 PCGs (Fig. 5). The comparative analysis of mt gene rearrangements in Arcidae reinforces the validity of our ML-tree and contributes new information on Arcidae phylogenetic relationships. As shown in Fig. 5, *T. kiyoni* was in a separate, more ancestral branch in the phylogenetic tree. Its gene order may represent the pleisomorphic gene arrangement in Arcidae. Hence, our analysis imply that the complicated gene rearrangement in mitochondrial genome could be considered as one of key characters in inferring higher-level phylogenetic relationship of Arcidae.

## Materials and Methods

### Sample collection and DNA extraction

Specimens of *T. kiyoni* and *P. pilula* were collected from the coastal water of Fujian Province, China. These samples were stored at −80 °C and deposited as voucher specimens (specimen number: TK01 and PP01) in Fisheries College, Ocean University of China. Each of the two Arcidae complete mitogenome sequenced was obtained from a single specimen. Total genomic DNA was extracted from adductor muscle by a modification of standard phenol-chloroform procedure as described by Li *et al*.^61^ and then stored at −20 °C.

### Determination of partial sequences

In order to design gene-specific primers, we first obtained partial *cox1* sequences for both *T. kiyoni* and *P. pilula*, with the universal primers of LCO1490/HCO2198^62^. Another short fragment, *rrnS* genes, were obtained from NCBI data base (GenBank accession no. JN974675 for *T. kiyoni* and JN974660 for *P. pilula*).

### Construction of BD GenomeWalker DNA libraries, PCR amplification and sequencing

Four BD GenomeWalker DNA libraries were constructed with the BD GenomeWalker Universal Kit (BD Biosciences, San Jose, CA, USA) following the manufacturer’s protocols.

The complete mitogenome of *T. kiyoni* and *P. pilula* were amplified using genome-walking based method, which involves two nested PCR reactions with a touch-down program modified from the BD GenomeWalker Universal Kit User Manual. The partial sequences of *cox1* and *rrnS* were used to design the initial sets of gene-specific primers, one (GSP1) for original PCR and the other (GSP2) for nested PCR, which were used for genome-walking to amplify both of the Arcidae mitogenome. The primer sequences used for genome-walking are presented in Supplementary Tables 3 and 4.

PCR was performed in a total volume of 50 μl including 2 U *Taq* DNA polymerase (TaKaRa, Dalian, China), about 100 ng template DNA, 1 μM forward and reverse primers, 200 μM of each dNTP, 1× PCR buffer and 2 mM MgCl_2_. The original PCR were carried out as follows using the outer adaptor primer1 (AP1) and outer gene-specific primer1 (GSP1): 10 s initial denaturation at 94 °C, 7 cycles of 30 s at 94 °C, 3 min at 72 °C, 32 cycles 30 s at 94 °C, 3 min at 67 °C, and 67 °C for an additional 7 min after the final cycle. A 1-μl sample of the original PCR was diluted in 59 μl of distilled water as the template for nested PCR amplification. The nested PCR were carried out as follows using the outer adaptor primer2 (AP2) and the outer, gene-specific primer2 (GSP2): 10 s initial denaturation at 94 °C, 5 cycles of 30 s at 94 °C, 3 min at 72 °C, 25 cycles 30 s at 94 °C, 3 min at 67 °C, and 67 °C for an additional 7 min after the final cycle. This procedure generally produces a single, major PCR product (100 bp–5000 bp) in at least one of the four libraries, which begins in a known sequence at the 5′ end of GSP2 and extend in the unkonwn adjacent genomic DNA.

PCR products were purified with EZ-10 spin column DNA gel extraction kit (Sangon Biotech), and then directly sequenced with the primer walking method. The sequencing was conducted on an ABI PRISM 3730 (Applied Biosystems) automatic sequencer in Beijing Genomics Institute (BGI) using standard Sanger sequencing chemistry.

### Sequencing assembling and annotation

All sequence data were analysed and arranged to create the full genomes using the Seqman program from DNASTAR (http://www.DNASTAR.com). The protein coding genes were analyzed with ORF Finder (http://www.ncbi.nlm.nih.gov/gorf/gorf.html) and BLASTx using the invertebrate mitochondrial genetic code. The tRNA genes were identified by ARWEN^63^ and DOGMA^64^ using the mito/chloroplast or invertebrate genetic code and the default search mode. The rRNA genes were identified by their similarity to published gene sequences and by using BLAST searches (http://www.ncbi.nlm.nih.gov/BLAST/).

The base composition and skewness analyses were performed and compared between *T. kiyoni* and *P. pilula* genomes, as well as the other four Arcidae genomes (*S. broughtonii* (46,985 bp)^3^, *S. kagoshimensis* (46,713 bp)^4^, *T. granosa* (31,589 bp)^14^ and *A. vellicata* (34,147 bp)^15^). The A + T content values were computed using Editseq program from DNASTAR. The GC and AT skews described strand bias were calculated according to the formulae by Perna and Kocher^65^, AT skew = (A − T)/(A + T); GC skew = (G − C)/(G + C), where A, T, G and C are the occurrences of the four nucleotides. The codon usage of each PCG were calculated using MEGA 5^66^. The ratios of nonsynonymous and synonymous substitutions rates (Ka/Ks) were estimated based on the Maximum-Likelihood (ML) method^67^ using KaKs_Calculator 2.0^68^ with the YN model.

The whole mitogenome sequence was tested for potentially tandem repeats by Tandem Repeats Finder 4.0^69^. Prediction of potential secondary structure was performed by the online version of the mfold software, version 3.2^70^, applying default settings. When multiple secondary structures were possible, the most stable (lowest free energy (ΔG)) was used.

The gene map of the *T. kiyoni* and *P. pilula* mitogenomes were generated with the program CGView^71^. The two mitochondrial genomes have been deposited in the GenBank database under the accession numbers KU975161 for *T. kiyoni* and KU975162 for *P. pilula*.

Predicted lengths of gene products and mitogenome sizes for up to 278 molluscs (see Supplementary Table 2). The statistical analysis was performed by using IBM SPSS Statistics 19 with Spearman rank correlations, as this test makes no assumption about the distribution of the data.

### Phylogenetic analyses

Along with mitochondrial genome sequence of *T. kiyoni* and *P. pilula*, all currently available mitochondrial genomes from Arcidae, including *S. broughtonii* (AB729113), *S. kagoshimensis* (KF750628), *T. granosa* (KJ607173) and *A. vellicata* (KP954700), were used in phylogenetic analysis.

The phylogenetic relationships were built based on the nucleotide sequences of 12 PCGs. *Crassostrea gigas* (AF177226) and *Crassostrea hongkongensis* (EU266073) from the family Ostreidae was used as outgroup. The twelve-partitioned nucleotide sequences of protein coding genes were aligned with MAFFT based on their nucleotide sequences using default settings^72^. The final nucleotide sequences of each gene were then concatenated into single contigs (6719 bp) for phylogenetic analyses. The best-fit nucleotide substitution models for each data partitions were selected by jModelTest^73^. We employed ML in RAxML Black-Box webserver (http://phylobench.vital-it.ch/raxml-bb/index.php)^74^ with GTR + G substitution model to each partition. For the ML analysis, 1000 bootstraps were used to estimate the node reliability.

## Additional Information

**How to cite this article**: Sun, S. *et al*. Complete mitochondrial genomes of *Trisidos kiyoni* and *Potiarca pilula*: Varied mitochondrial genome size and highly rearranged gene order in Arcidae. *Sci. Rep.* **6**, 33794; doi: 10.1038/srep33794 (2016).

## Supplementary Material

Supplementary Information

## Figures and Tables

**Table 1:** Organization of the mitochondrial genome of *Trisidos kiyoni*.

Gene	Strand	Position		Size		Codon		Intergenic nucleotides
		From	To	nt	aa	Start	Stop	
*cox1*	+	1	1560	1560	520	ATG	TAG	501
*trnA*	+	1594	1660	67				33
*trnH*	+	1685	1751	67				24
*nad1*	+	1922	2716	795	265	ATG	TAG	170
*cox2*	+	2806	3444	639	213	ATA	TAG	89
*trnI*	+	3454	3527	74				9
*nad5*	+	3562	5250	1689	563	ATA	TAA	34
*trnL*^*CUA*^	+	5301	5370	70				50
*Cytb*	+	5692	6630	939	313	ATG	TAA	321
*nad6*	+	6719	7024	306	102	ATG	TAA	88
*nad4l*	+	7292	7564	273	91	ATA	TAG	267
*nad4*	+	7591	8913	1323	441	ATG	TAA	26
*nad2*	+	9011	10168	1158	386	ATG	TAG	97
*trnF*	+	10179	10244	66				10
*trnS*^*AGA*^	+	11254	11320	67				1009
*trnK*	+	11348	11418	71				27
*trnP*	+	11442	11512	71				23
*trnR*	+	11525	11593	69				12
*trnW*	+	11598	11666	69				4
*trnD*	+	11729	11794	66				62
*rrnS*	+	12084	12793	710				289
*trnN*	+	12983	13053	71				189
*trnY*	+	13074	13140	67				20
*trnM*	+	13177	13247	71				36
*rrnL*	+	13335	14813	1479				87
*trnL*^*UUA*^	+	14833	14900	68				19
*trnC*	+	14930	15000	71				29
*trnQ*	+	15046	15112	67				45
*trnE*	+	15168	15235	68				55
*trnT*	+	15258	15324	67				22
*trnV*	+	15327	15395	69				2
*trnG*	+	15458	15526	69				62
*trnS*^*UCA*^	+	16525	16588	64				998
*cox3*	+	16679	17425	747	249	ATG	TAG	90
*atp6*	+	17901	18602	702	234	ATA	TAG	475
*nad3*	+	18698	19111	414	138	ATG	TAG	95

**Table 2:** Organization of the mitochondrial genome of *Potiarca pilula*.

Gene	Strand	Position		Size		Codon		Intergenic nucleotides
		From	To	nt	aa	Start	Stop	
*cox1*	+	1	1464	1464	488	ATA	TAA	2435
*nad5*	+	1434	3161	1728	576	ATG	TAA	−31
*trnM*	+	3172	3236	65				10
*nad1*	+	3240	4190	951	317	ATA	TTA	3
*nad4*	+	4217	5497	1281	427	ATA	TAA	26
*Cytb*	+	5609	6820	1212	404	ATA	TAG	111
*trnF*	+	6864	6931	68				43
*cox2*	+	7067	8008	942	314	ATA	TAA	135
*trnC*	+	8652	8716	65				643
*nad6*	+	9050	9370	321	107	ATG	TAA	333
*trnK*	+	9387	9458	72				16
*atp6*	+	9534	10178	645	215	ATG	TAG	75
*trnP*	+	10193	10259	67				14
*trnI*	+	10301	10367	67				41
*trnG*	+	10375	10445	71				7
*trnE*	+	11007	11075	69				561
*trnV*	+	11586	11651	66				510
*rrnL*	+	11689	13032	1344				37
*trnA*	+	13033	13100	68				0
*trnT*	+	13121	13189	69				20
*trnH*	+	13202	13270	69				12
*trnQ*	+	13283	13348	66				12
*nad3*	+	13495	13848	354	118	ATA	TAG	146
*nad4l*	+	13853	14122	270	90	ATA	TAG	4
*trnD*	+	14478	14546	69				355
*trnL*^*UUA*^	+	14632	14698	67				85
*trnY*	+	14769	14834	66				70
*trnN*	+	14852	14921	70				17
*rrnS*	+	15175	15847	673				253
*nad2*	+	16003	17070	1068	356	ATG	TAG	155
*trnS*^*AGA*^	+	17107	17174	68				36
*trnR1*	+	17179	17244	66				4
*trnL*^*CUA*^	+	17268	17335	68				23
*trnW*	+	17416	17488	73				80
*trnS*^*UCA*^	+	17496	17560	65				7
*cox3*	+	17547	18479	933	311	ATG	TAG	−14
*trnR2*	+	25888	25951	64				7408

**Table 3:** AT-content, AT-skew and GC-skew for mitochondrial genes of *Trisidos kiyoni* and *Potiarca pilula*.

Feature	(A + T)%		AT skew		GC skew	
	*T. kiyoni*	*P*. *pilula*	*T. kiyoni*	*P*. *pilula*	*T. kiyoni*	*P*. *pilula*
Whole genome	62.73	61.16	−0.30	−0.15	0.45	0.42
Protein-coding genes	61.63	60.36	−0.39	−0.36	0.43	0.42
*cox1*	60.13	60.93	−0.37	−0.32	0.29	0.28
*cox2*	61.50	57.01	−0.27	−0.09	0.36	0.29
*cox3*	60.11	59.16	−0.46	−0.28	0.38	0.42
*Cytb*	63.26	62.29	−0.39	−0.31	0.37	0.31
*nad1*	60.51	58.52	−0.38	−0.29	0.44	0.41
*nad2*	61.92	63.58	−0.36	−0.25	0.49	0.35
*nad3*	59.91	58.47	−0.44	−0.44	0.48	0.39
*nad4*	61.30	61.99	−0.43	−0.33	0.48	0.49
*nad4L*	60.44	62.22	−0.28	−0.25	0.69	0.63
*nad5*	62.58	58.80	−0.40	−0.19	0.47	0.52
*nad6*	62.75	61.68	−0.53	−0.28	0.58	0.66
*atp6*	64.72	60.78	−0.41	−0.26	0.51	0.43
tRNAs	58.38	53.45	−0.19	−0.07	0.35	0.30
*rrnS*	55.36	54.08	0.01	0.05	0.22	0.20
*rrnL*	62.61	61.46	−0.09	0.05	0.39	0.32
NCR	67.15	63.09	−0.25	−0.08	0.59	0.46

**Table 4:** Codon usage of *Trisidos kiyoni* and *Potiarca pilula* PCGs.

Amino acid	Code	N(RSCU)		Amino acid	Code	N(RSCU)	
		*T. kiyoni*	*P. pilula*			*T. kiyoni*	*P. pilula*
F (gaa)	TTT	349(1.59)	281(1.71)	Y (gta)	TAT	165(1.63)	129(1.61)
	TTC	89(0.41)	47(0.29)		TAC	38(0.37)	31(0.39)
L (taa)	TTA	76(1.82)	158(1.88)	Stop	TAA	64(0.85)	86(1.19)
	TTG	105(2.51)	160(1.90)		TAG	87(1.15)	59(0.81)
L (tag)	CTT	46(1.10)	79(0.94)	H (gtg)	CAT	17(1.21)	65(1.48)
	CTC	17(0.41)	20(0.24)		CAC	11(0.79)	23(0.52)
	CTA	3(0.07)	50(0.59)	Q (ttg)	CAA	7(0.70)	29(0.89)
	CTG	4(0.10)	38(0.45)		CAG	13(1.30)	36(1.11)
I (gat)	ATT	90(1.68)	121(1.66)	N (gtt)	AAT	106(1.64)	49(1.61)
	ATC	17(0.32)	25(0.34)		AAC	23(0.36)	12(0.39)
M (cat)	ATA	21(0.86)	71(0.84)	K (ttt)	AAA	35(0.80)	49(0.95)
	ATG	28(1.14)	99(1.16)		AAG	52(1.20)	54(1.05)
V (tac)	GTT	208(2.26)	130(1.55)	D (gtc)	GAT	87(1.67)	52(1.46)
	GTC	34(0.37)	29(0.35)		GAC	17(0.33)	19(0.54)
	GTA	56(0.61)	66(0.79)	E (ttc)	GAA	26(0.93)	65(0.95)
	GTG	70(0.76)	111(1.32)		GAG	30(1.07)	72(1.05)
S (tga)	TCT	65(1.22)	55(1.48)	C (gca)	TGT	185(1.50)	83(1.63)
	TCC	51(0.96)	19(0.51)		TGC	61(0.50)	19(0.37)
	TCA	75(1.41)	32(0.86)	W (tca)	TGA	90(0.66)	90(0.88)
	TCG	44(0.83)	16(0.43)		TGG	181(1.34)	115(1.12)
P (tgg)	CCT	11(1.05)	46(1.67)	R (tcg)	CGT	10(0.83)	42(1.68)
	CCC	18(1.71)	21(0.76)		CGC	7(0.58)	8(0.32)
	CCA	10(0.95)	25(0.91)		CGA	6(0.50)	15(0.60)
	CCG	3(0.29)	18(0.65)		CGG	25(2.08)	35(1.40)
T (tgt)	ACT	9(0.88)	41(1.91)	S (tct)	AGT	60(1.13)	43(1.15)
	ACC	11(1.07)	8(0.37)		AGC	34(0.64)	13(0.35)
	ACA	14(1.37)	24(1.12)		AGA	35(0.66)	41(1.10)
	ACG	7(0.68)	13(0.60)		AGG	61(1.15)	79(2.12)
A (tgc)	GCT	39(1.46)	58(1.97)	G (tcc)	GGT	139(1.62)	136(1.42)
	GCC	28(1.05)	10(0.34)		GGC	59(0.69)	18(0.19)
	GCA	25(0.93)	22(0.75)		GGA	51(0.59)	70(0.73)
	GCG	15(0.56)	28(0.95)		GGG	95(1.10)	159(1.66)

N: number of occurrence of the codon.

RSCU: relative synonymous codon usage.
