# Supplementary material for: Complete mitochondrial genomes of Trisidos kiyoni and Potiarca pilula: Varied mitochondrial genome size and highly rearranged gene order in Arcidae
Source: Sci Rep. 2016 Sep 22;6:33794. doi: 10.1038/srep33794 (PMC5031957; doi:10.1038/srep33794)

Complete mitochondrial genomes of *Trisidos kiyoni* and *Potiarca pilula*: Varied mitochondrial genome size and highly rearranged gene order in the family Arcidae

Shao'e Sun, Qi Li*, Lingfeng Kong, Hong Yu

Key Laboratory of Mariculture, Ministry of Education, Ocean University of China, Qingdao 266003, China

*Corresponding author: Tel: +8653282031622. Fax: +8653282032773.
E-mail: qili66@ouc.edu.cn

Supplementary Table 1. The ratio of nonsynonymous and synonymous substitutions (Ka/Ks) estimated with KaKs_Calculator in all 12 protein genes of the four Arcidae species, *Scapharca broughtonii* (Sb), *Scapharca kagoshimensis* (Sk), *Tegillarca granosa* (Tg), *Anadara vellicata* (Av), *Trisidos kiyoni* (Tk) and *Potiarca pilula* (Pp).

	cox1	*cox2*	*cox3*	*Cytb*	*atp6*	*nad1*	*nad2*	*nad3*	*nad4*	*nad4L*	*nad5*	*nad6*
Sb-Sk	0.0549	0.0462	0.1536	0.2183	0.0972	0.0924	0.1041	0.1422	0.1024	0.0537	0.0715	0.0963
Sb-Tg	0.0274	0.0944	0.0873	0.0470	0.1527	0.0664	0.1575	0.1960	0.0713	0.3641	0.1340	0.1565
Sb-Av	0.0207	0.0821	0.0308	0.0019	0.2461	0.0456	0.8421	0.0375	0.0910	0.2935	0.1533	0.1889
Sk-Tg	0.0315	0.0885	0.0448	0.0033	0.1754	0.0934	0.1039	0.1989	0.0495	0.2443	0.0629	0.2127
Sk-Av	0.0226	0.1060	0.0599	0.0048	0.0945	0.0559	0.9913	0.3161	0.1101	0.1114	0.1029	0.1861
Tg-Av	0.0212	0.1601	0.1237	0.0444	0.1619	0.0674	0.9878	0.0644	0.1242	0.1186	0.1467	0.1428
Tk-Av	0.0478	0.1348	0.0884	0.0035	0.1524	0.4086	0.6276	0.2974	0.1300	0.7841	0.1149	0.9418
Tk-Sb	0.0511	0.1277	0.0977	0.0786	0.1624	0.1438	0.2402	0.1295	0.1305	0.4202	0.1224	0.4566
Tk-Sk	0.0568	0.1336	0.1229	0.1107	0.2032	0.2278	0.2265	0.1934	0.1331	0.2716	0.1066	0.2177
Tk-Tg	0.0506	0.1336	0.0921	0.0747	0.1985	0.1414	0.2202	0.2028	0.1217	0.2631	0.1197	0.2093
Pp-Av	0.0166	0.1133	0.0512	0.0522	0.2136	0.2533	0.9272	0.0530	0.1524	0.4602	0.1264	0.1754
Pp-Sb	0.0229	0.0953	0.0913	0.0454	0.1961	0.1743	0.1508	0.0569	0.0985	0.2411	0.1682	0.1700
Pp-Tk	0.0516	0.1232	0.1036	0.0707	0.1746	0.2024	0.2285	0.1106	0.1145	0.6204	0.1972	0.3723
Pp-Sk	0.0273	0.1363	0.0836	0.0882	0.2160	0.1423	0.2343	0.1001	0.1323	0.3164	0.0663	0.2452
Pp-Tg	0.0239	0.1452	0.1012	0.0436	0.1679	0.1313	0.1096	0.0767	0.3541	0.3369	0.1905	0.1655

Supplementary Table 2. The 256 molluscs included in the analysis of correlation between mitochondrial genome size with gene lengths.

Species	Accession number	Mitogenome sizes	Gene lengths		
			CO1-3	*Cytb*	*rrnS and rrnL*
Scapharca broughtonii	AB729113	46985	3009	1278	1997
Fulvia mutica	AB809077	19110	3114	1131	2057
Crassostrea gigas	AF177226	18224	2955	1119	2146
Venerupis philippinarum	AB065375	22676	3907	1248	2657
Mizuhopecten yessoensis	AB271769	20414	3480	1161	2385
Laternula elliptica	KF534717	14622	3091	1137	1962
Solenaia oleivora	KF296320	16392	2706	1161	2130
Ostrea lurida	KC768038	16344	3094	1053	2229
Atrina pectinata	KC153059	16811	3063	1146	2251
Perna viridis	JQ970425	16014	3051	1152	1935
Solen strictus	JN786377	16535	3135	1149	2196
Meretrix petechialis	EU145977	19567	3723	1218	2768
Anodonta anatina	KF030964	15653	3003	1155	2127
Mimachlamys senatoria	KF214684	17383	3204	1263	2326
Lamprotula tortuosa	KC109779	15722	3003	1149	2140
Cristaria plicata	FJ986302	15712	3003	1149	2129
Coelomactra antiquata	JQ423460	17199	3447	1281	2194
Placopecten magellanicus	DQ088274	32115	3025	1188	2357
Mytilus californianus	GQ527172	16730	3321	1308	2191
Sinanodonta woodiana	HQ283346	16242	3003	1149	2131
Sinonovacula constricta	EU880278	17225	3339	1146	2142
Chlamys farreri	EU715252	21695	3243	1173	2390
Crassostrea hongkongensis	EU266073	16475	3192	1206	2302
Crassostrea virginica	AY905542	17244	3189	1212	2456
Ostrea edulis	JF274008	16320	3147	1161	2159
Saccostrea mordax	FJ841968	16532	3171	1173	2319
Mimachlamys nobilis	FJ415225	17963	3048	1077	2334
Crassostrea iredalei	FJ841967	22446	3195	1275	2262
Crassostrea sikamea	FJ841966	18242	3195	1236	2320
Crassostrea angulata	FJ841965	18408	3195	1239	2265
Crassostrea ariakensis	FJ841964	18425	3183	1215	2265
Mytilus trossulus	GU936625	18653	3321	1308	2191
Hyriopsis schlegelii	HQ641406	15939	3003	1155	2130
Solen grandis	HQ703012	16784	3141	1149	2185
Hiatella arctica	DQ632742	18244	3565	1155	2348

Crassostrea nippona	HM015198	20030	3180	1122	2393
Argopecten irradians	EU023915	16221	3129	1146	2252
Pinctada margaritifera	HM467838	15680	3142	1146	1961
Pinctada maxima	GQ452847	16994	3159	1176	2054
Mytilus edulis	AY484747	16740	3178	1194	2189
Mytilus galloprovincialis	AY497292	16744	3187	1308	2191
Musculista senhousia	GU001954	20612	3252	1197	2769
Paphia euglypta	GU269271	18643	3837	1194	2669
Meretrix lamarckii	GU071281	21209	3924	1248	2584
Meretrix meretrix	GQ463598	19826	3723	1218	2768
Arctica islandica	KF363951	18289	3624	1122	2135
Paphia undulata	JF969278	18154	3918	1161	2414
Paphia textile	JF969277	18561	3927	1164	2506
Paphia amabilis	JF969276	19629	3900	1218	2588
Meretrix lusoria	GQ903339	20268	3807	1227	2470
Lucinella divaricata	EF043342	18940	3282	1143	2021
Loripes lacteus	EF043341	17321	3234	1140	2027
Acanthocardia tuberculata	DQ632743	16104	3153	1158	2037
Solecurtus divaricatus	JN398367	16749	3486	1248	2267
Semele scabra	JN398365	17117	3792	1176	2191
Nuttallia olivacea	JN398364	18182	3552	1242	2295
Soletellina diphos	JN398363	16352	3564	1233	2219
Moerella iridescens	JN398362	16799	3399	1233	2131
Meretrix lyrata	KC832317	21625	3762	1188	2526
Lampsilis ornata	AY365193	16060	3003	1149	2161
Hyriopsis cumingii	HM347668	15957	3003	1155	2117
Solenaia carinatus	KC848654	16716	2487	1161	2153
Toxolasma parvus	HM856639	15949	3003	1149	2176
Lasmigona compressa	HM856638	15903	3003	1149	2123
Utterbackia imbecillis	HM856637	16103	3003	1152	2114
Utterbackia peninsularis	HM856635	16803	3582	1161	2153
Margaritifera falcata	HM856634	16121	3006	1143	2181
Unio pictorum	HM014130	15760	3015	1149	2171
Pyganodon grandis	FJ809754	15848	3000	1146	2118
Venustaconcha ellipsiformis	FJ809752	17174	3543	1167	2149
Quadrula quadrula	FJ809750	16033	3003	1152	2154
Solemya velum	JQ728447	15660	3003	1122	2261
Tegillarca granosa	KJ607173	31589	3540	1221	2085
Scapharca kagoshimensis	KF750628	46713	2778	1080	2058
Lamprotula coreana	JX050180	15697	3000	1149	2142

Unio douglasiae	KM657954	15767	3018	1140	2161
Mactra chinensis	KJ754823	17285	3453	1290	2034
Panopea generosa	KM580067	15585	3090	1158	2092
Panopea globosa	KM580068	15469	3099	1158	2103
Brachidontes exustus	KM233636	16600	3168	1206	2091
Mytilus coruscus	KJ577549	16642	3321	1308	2189
Mya arenaria	KJ755996	17947	4050	1041	2389
Dahurinaia dahurica	KF514426	16112	3006	1143	2174
Arconaia lanceolata	KJ144818	15782	3018	1023	2092
Lamprotula gottschei	KJ018924	15915	3009	1149	2202
Lamprotula leai	JQ691662	16530	3009	1149	2138
Lutraria rhynchaena	HG799089	16927	3666	1248	2307
Argopecten purpuratus	KF601246	16266	3006	1158	2220
Saccostrea cucullata	KP967577	16396	3183	1110	2337
Crassostrea gasar	KR856227	17685	3204	944	2455
Anodonta arcaeformis	KF667530	15672	3006	1149	2120
Anodonta lucida	KF667529	16285	3003	1149	2120
Perna perna	KM655841	18415	3270	1158	2005
Tridacna squamosa	KP205428	20930	3286	1219	2101
Anadara vellicata	KP954700	34147	2823	1113	1876
Trisidos kiyoni	KU975161	19614	2946	939	2451
Potiarca pilula	KU975162	27895	3339	1212	2017
Anodonta euscaphys	KP187851	15741	3003	1149	2122
Potomida littoralis	KT247374	15789	3009	1149	2230
Leptodea leptodon	KT723012	16133	3024	1159	2228
Lanceolaria grayana	KJ495725.	15736	2997	1149	2130
Cuneopsis pisciculus	KP273584	15907	3009	1149	2180
Saxidomus purpuratus	KP419933	19637	3435	1206	2519
Calyptogena magnifica	KR862368	19738	3849	1134	2154
Argopecten ventricosus	KT161261	16079	3129	1159	2199
Limnoperna fortunei	KP756905	18145	2853	1134	1940
Haliotis rubra	AY588938	16907	3018	1140	2558
Haliotis laevigata	KJ472483	16545	3030	1140	2477
Haliotis discus	KF724723	16886	3018	1140	2564
Haliotis diversicolor	HQ832672	16266	3018	1140	2452
Haliotis tuberculata	FJ605487	15930	3018	1140	2573
Haliotis tuberculata tuberculata	FJ599667	16521	3018	1140	2604
Tegula lividomaculata	KT207826	17375	3012	1140	2641
Tegula brunnea	JN790613	17690	3012	1152	2628
Bolma rugosa	KT207824	17432	3006	1140	2670

Angaria neglecta	KR297248	19470	3009	1140	2801
Lunella aff. cinerea	KF700096	17670	3009	1140	2402
Lepetodrilus nux	LC107880	16353	3033	1137	2506
Lepetodrilus schrolli	KR297250	15579	3033	1137	2483
Granata lyrata	KR297249	17632	3078	1137	2671
Diodora graeca	KT207825	17209	3006	1140	2526
Fissurella volcano	JN790612	17575	2925	1143	2420
Chrysomallon squamiferum	AP013032	15388	3015	1140	2221
Phasianella solida	KR297251	16698	3024	1140	2460
Nerita versicolor	KF728890	15866	3015	1137	2168
Nerita melanotragus	GU810158	15261	3018	1137	2172
Nerita tessellata	KF728889	15741	3009	1137	2169
Nerita fulgurans	KF728888	15343	3018	1137	2167
Aplysia kurodai	KF148053	14131	2994	1122	1751
Aplysia californica	AY569552	14117	3042	1104	1751
Aplysia dactylomela	DQ991927	14128	3012	1125	1751
Roboastra europaea	AY083457	14472	2985	1122	1849
Notodoris gardineri	DQ991934	14424	2982	1143	1792
Melibe leonina	KP764764	14513	2982	1134	1888
Salinator rhamphidia	JN620539	14007	2976	1113	1739
Siphonaria gigas	JN627205	14518	3012	1125	1864
Siphonaria pectinata	AY345049	14065	2976	1113	1715
Ascobulla fragilis	AY345022	14745	2994	1122	1833
Elysia chlorotica	EU599581	14132	3003	1122	1750
Onchidella celtica	AY345048	14150	2988	1122	1764
Trimusculus reticulatus	JN632509	14044	2973	1110	1776
Pyramidella dolabrata	AY345054	13856	2958	1113	1693
Pupa strigosa	AB028237	14189	3009	1128	1798
Micromelo undata	DQ991933	14160	2994	1125	1791
Naticarius hebraeus	KP716634	15384	3027	1140	2403
Strombus gigas	KM245630	15461	3003	1140	2366
Galeodea echinophora	KP716635	15388	3003	1140	2361
Cymatium parthenopeum	EU827200	15270	3003	1140	2324
Eualetes tulipa	HM174254	15078	3006	1140	2213
Dendropoma maximum	HM174253	15578	2994	1140	2480
Dendropoma gregarium	HM174252	15641	3003	1149	2328
Thylacodes squamigerus	HM174255	15544	3012	1140	2484
Buccinum pemphigus	KT962044	15265	3003	1140	2146
Babylonia lutosa	KF897830	15346	3000	1140	2311
Babylonia areolata	HQ416443	15445	3000	1140	2148

Volutharpa perryi	KT382829	15255	3003	1140	2238
Varicinassa variciferus	KM603509	15269	3003	1152	2309
Nassarius reticulatus	EU827201	15271	3003	1140	2314
Cancellaria cancellata	EU827195	16648	3012	1140	2441
Conus tribblei	KT199301	15570	3015	1140	2322
Conus tulipa	KR006970	15756	3015	1140	2322
Conus textile	DQ862058	15562	3015	1140	2336
Conus consors	KF887950	16112	3015	1140	2316
Conus borgesi	EU827198	15536	3015	1140	2321
Fusiturris similis	EU827197	15595	3009	1140	2309
Lophiotoma cerithiformis	DQ284754	15380	3012	1140	2338
Terebra dimidiata	EU827196	16513	3000	1140	2352
Rapana venosa	KM213962	15271	3000	1140	2330
Concholepas concholepas	JQ446041	15495	2907	1140	2332
Reishia clavigera	DQ159954	15285	2997	1140	2322
Bolinus brandaris	EU827194	15380	3000	1140	2343
Cymbium olla	EU827199	15375	3006	1140	2296
Amalda northlandica	GU196685	15354	3000	1140	2264
Turritella bacillum	KU221394	15868	3003	1140	2306
Semisulcospira libertina	KF736848	15432	3000	1140	2298
Ifremeria nautilei	KC757644	15664	3003	1140	2400
Lottia digitalis	DQ238599	26835	3168	1170	2524
Galba pervia	JN564796	13768	2958	1098	1722
Radix balthica	KP098541	13983	2928	1065	1686
Radix auricularia	KP098540	13745	2940	1080	1700
Radix swinhoei	KP279638	14241	2925	1119	2197
Physella acuta	JQ390526	14314	2925	1119	2023
Planorbarius corneus	KP279639	13687	2949	1083	1531
Biomphalaria glabrata	AY380567	13670	2964	1101	1689
Biomphalaria tenagophila	EF433576	13722	2985	1101	1689
Cipangopaliduna cathayensis	KM503121	17157	3012	1140	2282
Marisa cornuarietis	KM100140	15923	3003	1140	2336
Pomacea canaliculata	KJ739609	15707	3003	1140	2263
Pomacea maculata	KR350466	15516	3003	1140	2265
Oncomelania hupensis robertsoni	EU079378	15191	3003	1140	2289
Oncomelania hupensis hupensis	EU001660	15186	3003	1140	2287
Oncomelania hupensis	FJ997214	15182	3003	1140	2282
Tricula hortensis	EU440735	15179	3003	1140	2277
Potamopyrgus estuarinus	GQ996415	15120	3003	1140	2278
Potamopyrgus antipodarum	GQ996430	15110	3003	1140	2278

Cerion incanum	KM365085	15177	3027	1125	1964
Achatina fulica	KJ744205	15057	3006	1125	1821
Mastigeulota kiangsinensis	KM083123	14029	2982	1125	1694
Dolicheulota formosensis	KR338956	14237	2982	1122	1724
Aegista aubryana	KT192071	14238	3012	1137	1700
Aegista diversifamilia	KR002567	14039	3012	1134	1700
Helix aspersa	JQ417194	14050	2976	1098	1692
Cylindrus obtusus	JN107636	14610	3027	1128	1697
Camaena cicatricosa	KM365408	13843	2997	1113	1679
Succinea putris	JN627206	14092	2982	1107	1775
Naesiotus nux	KT821554	15197	3015	1119	1374
Albinaria caerulea	NC_001761	14130	2985	1104	1794
Vertigo pusilla	KC185405	14078	2976	1113	1772
Pupilla muscorum	KC185404	14149	2976	1110	1796
Gastrocopta cristata	KC185403	14060	2976	1113	1787
Platevindex mortoni	GU475132	13991	2970	1101	1737
Peronia peronii	JN619346	13968	2973	1110	1747
Rhopalocaulis grandidieri	JN619347	14523	2937	1068	1722
Pedipes pedipes	JN615140	16708	2988	1110	1924
Ovatella vulcani	JN615139	14274	2949	1110	1768
Myosotella myosotis	JN606067	14215	2946	1110	1755
Sepia apama	AP013073	16184	3000	1140	2219
Sepia esculenta	AB266516	16199	3000	1140	2276
Sepia latimanus	AP013074	16225	3000	1140	2243
Sepia lycidas	KJ162574	16228	3000	1140	2255
Sepia officinalis	AB240155	16163	2997	1140	2250
Sepia pharaonis	KC632521	16208	3000	1140	2277
Sepia aculeata	KF690633	16219	3000	1140	2276
Sepiella inermis	KF040369	16191	2997	1140	2259
Sepiella japonica	AB675082	16172	2997	1140	2264
Sepiella maindroni	KR912215	16170	2997	1140	2263
Idiosepius sp.	KF647895	16183	3003	1155	2290
Semirossia patagonica	AP012226	17086	3000	1140	2215
Loligo opalescens	GQ225110	17387	3009	1140	2340
Loligo bleekeri	AB029616	17211	3006	1140	2084
Sepioteuthis lessoniana	KM878671	16694	3003	1140	1909
Doryteuthis opalescens	KP336703	17370	3009	1140	2340
Loliolus beka	KT254309	17483	3006	1140	2370
Loliolus uyii	KP265013	17134	3006	1140	2364
Uroteuthis chinensis	KT362380	17353	3006	1140	2393

Uroteuthis duvaucelii	KR051264	17413	3006	1140	2390
Uroteuthis edulis	AB675080	17360	3006	1140	2349
Architeuthis dux	KC701763	20332	3000	1140	2323
Bathyteuthis abyssicola	AP012225	20075	3000	1140	2151
Watasenia scintillans	KJ845633	20089	3000	1140	2262
Ommastrephes bartramii	AB715401	20308	3003	1140	2384
Dosidicus gigas	EU068697	20324	3003	1140	2403
Sthenoteuthis oualaniensis	EU660576	20306	3003	1140	2413
Todarodes pacificus	AB240153	20247	3009	1146	2378
Illex argentinus	KP336702	20278	3003	1146	2392
Amphioctopus aegina	KT428877	15545	3033	1146	2264
Amphioctopus fangsiao	AB240156	15979	3000	1140	2313
Cistopus chinensis	KF017606	15706	3000	1107	2327
Cistopus taiwanicus	KF017605	15793	3000	1131	2401
Octopus bimaculatus	KT581981	16084	3000	1140	2262
Octopus conispadiceus	KJ789854	16027	3000	1140	2365
Octopus minor	HQ638215	15974	3000	1140	2380
Octopus vulgaris	AB158363	15744	3000	1140	2337
Vampyroteuthis infernalis	AB266515	15617	2997	1140	2186
Nautilus macromphalus	DQ472026	16258	2997	1134	2239
Allonautilus scrobiculatus	KP892752	16132	2997	1134	2231

Supplementary Table 3. Primers used in the genome walking PCRs for amplification of fragments in *Trisidos kiyoni* mitochondrial genome.

Pairs	Sequence (5'-3')	Position	Amplification direction
1TACO1SP1	TCACTAAACCGAACCCTGGCAAAATAAG	733-760	Counterclockwise
1TACO1SP2	AACATTCTTCAAGCAAAAATTGGCAACC	536-563	Counterclockwise
2TCO1SP1	GGTATTTTAGGGTTTGTTGTTTGGGCG	838-864	Clockwise
2TCO1SP2	TGATAGTCGTGCTTATTTCACTGGGGCT	894-921	Clockwise
3TCO1SP1	AGGATGATGGTGATAGTTGTTGGGGATTT	2666-2694	Clockwise
3TCO1SP2	TTCTGGGTTAGGGTTGTAGTGAAAAATGTG	2698-2727	Clockwise
4TCO1SP1	AGGCTGGTTTGTGGTCATTGAAGGTTG	4006-4032	Clockwise
4TCO1SP2	GCTGGGTGTGGGGTAATGTTGTGTTT	4042-4067	Clockwise
5TCO1SP1	TTGGGCTGAGGGAGCAGTGTATTGAT	5045-5070	Clockwise
5TCO1SP2	GAGAAGTCCATTGTCGTGCTCAAAGGTT	5138-5165	Clockwise
6TCO1SP1	CCATAACATGAGGATGGTATGTTCTCACCC	5337-5366	Clockwise
6TCO1SP2	GTTATATTTGGGAGGTGGATGAAAGTTGTC	5440-5469	Clockwise
7TCO1SP1	TTAAGGTGGCTCGGAAGTTGTGCTGT	6487-6512	Clockwise
7TCO1SP2	ATTATGTGATGGAGTGGAATCGGGAGT	6602-6628	Clockwise
8TA12SSP1	CCTACCACTCCTGTCCCTTTTACCGC	9258-9283	Counterclockwise
8TA12SSP2	AAAACCACAGAGAGAACCATCCCAACAG	9150-9177	Counterclockwise
9TA12SSP1	GACTACCTCAGCACCTTCAACGCCAC	10201-10226	Counterclockwise
9TA12SSP2	GCCCCCCACTAACAAACAATCAAATCTAT	10148-10176	Counterclockwise
10TA12SSP1	CTACCCACACTCTATCAGCTTTGAAGGC	11657-11684	Counterclockwise
10TA12SSP2	TAAAACTTTCCCCCCACACATAAACCATT	11624-11652	Counterclockwise
11T12SSP1	GCATGGTAGAAGAAATTCGCAGAAGC	11879-11904	Clockwise
11T12SSP2	GTTGGGTGTAATCAAGTAAGTCAATATCAG	12187-12216	Clockwise
12TA12SSP1	CCAAACGAACCATGCCACATTCCTTA	12495-12520	Counterclockwise
12TA12SSP2	GCTGACAACGACCATACACCAACTGATAC	12449-12477	Counterclockwise
13T12SSP1	TGTTGAATGTGGTTTACCTTTGGCTGC	14577-14603	Clockwise
13T12SSP2	CAGGTCAAAAGGGTGGGACTGTTCGT	14606-14631	Clockwise
14T12SSP1	CCATTTGTTTTGTGGTGGAATGAGGTT	15503-15529	Clockwise
14T12SSP2	TTTATCGTGTGTTTTTGGATTGGGCTG	15640-15666	Clockwise
15TACO1SP1	CTTCCCTTCACTCATACCACAACCCAAC	16811-16838	Counterclockwise
15TACO1SP2	AGCAATAAACGCCCCACACAAACTTC	16729-16754	Counterclockwise
16TACO1SP1	CCTAACACAACATTTCCCATCAACCGAG	17399-17426	Counterclockwise
16TACO1SP2	GGACACCCCTCAGGAACAAAACTACCC	17339-17365	Counterclockwise
17TACO1SP1	TAAAACTTTCCCCCCACACATAAACCATT	18075-18102	Counterclockwise
17TACO1SP2	TCAAAACCCAACCACAATAACCAGCC	17965-17990	Counterclockwise
18TACO1SP1	TAAGAACCATATCACCCGCCCCAAAT	18379-18404	Counterclockwise

18TACO1SP2	AACATCCACAAAATGTCAATACCACACTGC	18281-18310	Counterclockwise
19TACO1SP1	GCTGACCACCAAAAGTTGACTCACGAAT	19058-19085	Counterclockwise
19TACO1SP2	ACAAGAACTAACACCCGACCATCCATTT	18996-19023	Counterclockwise

Supplementary Table 4. Primers used in the genome walking PCRs for amplification of fragments in *Potiarca pilula* mitochondrial genome.

Pairs	Sequence (5'-3')	Position	Amplification direction
1PACO1SP1	CAAAAAACAGGCATTCGCTCTGGGAT	382-407	Counterclockwise
1PACO1SP2	TTCACCTAGATAAAGGCGGGTATAAAGTCC	230-259	Counterclockwise
2PCO1SP1	TCCCAGAGCGAATGCCTGTTTTTTGT	383-408	Clockwise
2PCO1SP2	TCGGGGTGTTAGGGTTTATTGTTTGAGC	692-719	Clockwise
3PCO1SP1	GGGTTTTAGGTCTGTGAGTAGTGGGGTG	1510-1537	Clockwise
3PCO1SP2	GGGGTGAGGGAGGATGTGCTTAGAAAT	1532-1558	Clockwise
4PCO1SP1	GTTGGGTTTAGGTCAGTTAGACGGAGGG	2622-2649	Clockwise
4PCO1SP2	GGTCACTTCAAGGCGGTATCGGTATG	2874-2899	Clockwise
5PCO1SP1	AGTCTATGGGCTCATAACCTGGAAATACG	3190-3218	Clockwise
5PCO1SP2	TGTCGGTTGCTTTTTTTACTTTGGTGG	3313-3340	Clockwise
6PCO1SP1	GGGGTTCTTATTAGGGGATGAGCGTCT	3612-3638	Clockwise
6PCO1SP2	AGGGATGGGTGTGAACTGCTTTTACG	3775-3800	Clockwise
7PCO1SP1	TTGTTGGGGGGATAAAGGGAAAAAGTT	4410-4436	Clockwise
7PCO1SP2	GCATTTAGAAGTAGGAGGGTTGTGGCG	4478-4504	Clockwise
8PCO1SP1	GGTGGTGATTATTTCTTGCCCCATTTC	4707-4733	Clockwise
8PCO1SP2	GCTGGTTCAATGATTTTGGCTGGTTT	4799-4824	Clockwise
9PA16SP1	TTAACAAAAACCAAGCGACACTTACCCCT	6310-6338	Counterclockwise
9PA16SP2	TTCCACGAATAATAAGGGTGAAAAGGC	6274-6300	Counterclockwise
10PA16SP1	CATACCGCTTGTTTATTGTTTCATACTCCC	7359-7388	Counterclockwise
10PA16SP2	AATCACCTTCACATCCAACACCGTCC	7317-7342	Counterclockwise
11PA16SP1	CTTACCCCTTCAACTCACCTTTTCCCTC	8101-8128	Counterclockwise
11PA16SP2	TTATTTTTTACTTGGTGGTGGGCACATTC	7980-8008	Counterclockwise
12PA16SP1	CCTCAACTACAAAACCACCACCTTCCAG	8294-8322	Counterclockwise
12PA16SP2	CCTTACCTCTAAAACCTCCCCGCAAC	8250-8276	Counterclockwise
13PA16SP1	CACCGATAAAAAAAGCAATAATCCACCCT	8946-8974	Counterclockwise
13PA16SP2	AGAGAACCACCCAGAAAAACAACAAATCC	8741-8769	Counterclockwise
14PA16SP1	TCTTTCCTCATCCCACCACCCACAAT	10475-10500	Counterclockwise
14PA16SP2	ACCTTCCCCAGTAGACCTGACGCTCC	10224-10249	Counterclockwise
15PA16SP1	ATAAACACCCGAAAACTAAACTCACCCCT	11094-11122	Counterclockwise
15PA16SP2	TCCTACTTCCACCACCACACTTTTACAGC	10838-10866	Counterclockwise
16PA16SP1	TCATTCTGGAGACCGCAGTTAAGCCTAC	12136-12163	Counterclockwise
16PA16SP2	CTATGACCTCTGTAGCCTCCTTGCTGGT	11927-11954	Counterclockwise
17PA12SP1	CCGCCTCACACACCCTTAAAACCAAT	14014-14039	Counterclockwise
17PA12SP2	CCAAAGACCCCTCTCTCCACTCAAACC	13811-13837	Counterclockwise
18PA12SP1	TGCCACCCCAGCACATAACCTCAACT	14756-14781	Counterclockwise
18PA12SP2	TTCTCCCTGCCCCGACCAAATCTTAC	14724-14749	Counterclockwise

19PA12SP1	GTCAAGTCTCACGGGCTGGTTCATCG	15455-15480	Counterclockwise
19PA12SP2	CTTTTACCTCCATCCGACGTTGTTGC	15408-15433	Counterclockwise
20P12SSP1	CGATGAACCAGCCCGTGAGACTTGAC	15455-15480	Clockwise
20P12SSP2	AATGAAAACAAATCAAACGGAGTCGGG	15663-15689	Clockwise
21P12SSP1	TGGGGGGATGCTTTTGCGTTGACTAC	16381-16406	Clockwise
21P12SSP2	GAAAGGGGGTGATATTATGTTTGTTGTGG	16453-16481	Clockwise
22P12SSP1	TTTGTGCTGTGGTGTTAGGGGGGTATG	16919-16945	Clockwise
22P12SSP2	AATGTCTTGGTTGGCTTTGTGAGGATGTT	17017-17045	Clockwise
23P12SSP1	TGTGGGTGGGGTTTATCCTTTTTGTATG	17824-17851	Clockwise
23P12SSP2	AGTTGGGGTGTGTGTATCCTCCTGTCG	17920-17946	Clockwise
24P12SSP1	CTAGGGGTTTGTGTGATTTTTTTTGCG	19225-19251	Clockwise
24P12SSP2	TGGAAGAAGGGGTAGAAGGAGTTTTGTTT	19430-19458	Clockwise
25P12SSP1	GGTGGGGTTTAGTTTTCTTGACGGTATTC	19725-19753	Clockwise
25P12SSP2	ATTGAAGGGAGGGAGAAGAAAAAGTGATT	19951-19979	Clockwise
26P12SSP1	TCAAATGAAATCAGGTAAGGCTCTCTATGG	21321-21350	Clockwise
26P12SSP2	AACAGATCATGCCACAATGAGTATAAGAAC	21500-21528	Clockwise
27PACO1SP1	GAAGCAACACATCAATAAGCCAACCG	22677-22702	Counterclockwise
27PACO1SP2	CACAAACCCAACCCTCCCTACAACAT	22527-22552	Counterclockwise
28PACO1SP1	GCAATCTTCACGCACTACCCTTTTTATC	24281-24308	Counterclockwise
28PACO1SP2	TTACCAACCGCCTGACAATTTGCCAC	24186-24211	Counterclockwise
29PACO1SP1	AAACTCTTCCCTCCCTTCCTCTCAGC	24643-24668	Counterclockwise
29PACO1SP2	GGTAAACAAGCCTCCTCCCCAAAAGT	24567-24592	Counterclockwise
30PACO1SP1	CACCGAAGACTCTCCAAGTAAAGCCCT	26187-26213	Counterclockwise
30PACO1SP2	CTGAAGAAGAAGTTACCTCAGCCAAATCGT	26095-26124	Counterclockwise
31PACO1SP1	CCTCTCGATAAGCTCGCCTAGACATTGT	26976-27003	Counterclockwise
31PACO1SP2	CTCTAAATCGGCATCACTTAGCCTACCC	26834-26861	Counterclockwise

Supplementary Fig 1. Cloverleaf structures of the 22 mt tRNA genes deduced from the mitochondrial genome of *Trisidos kiyoni*.

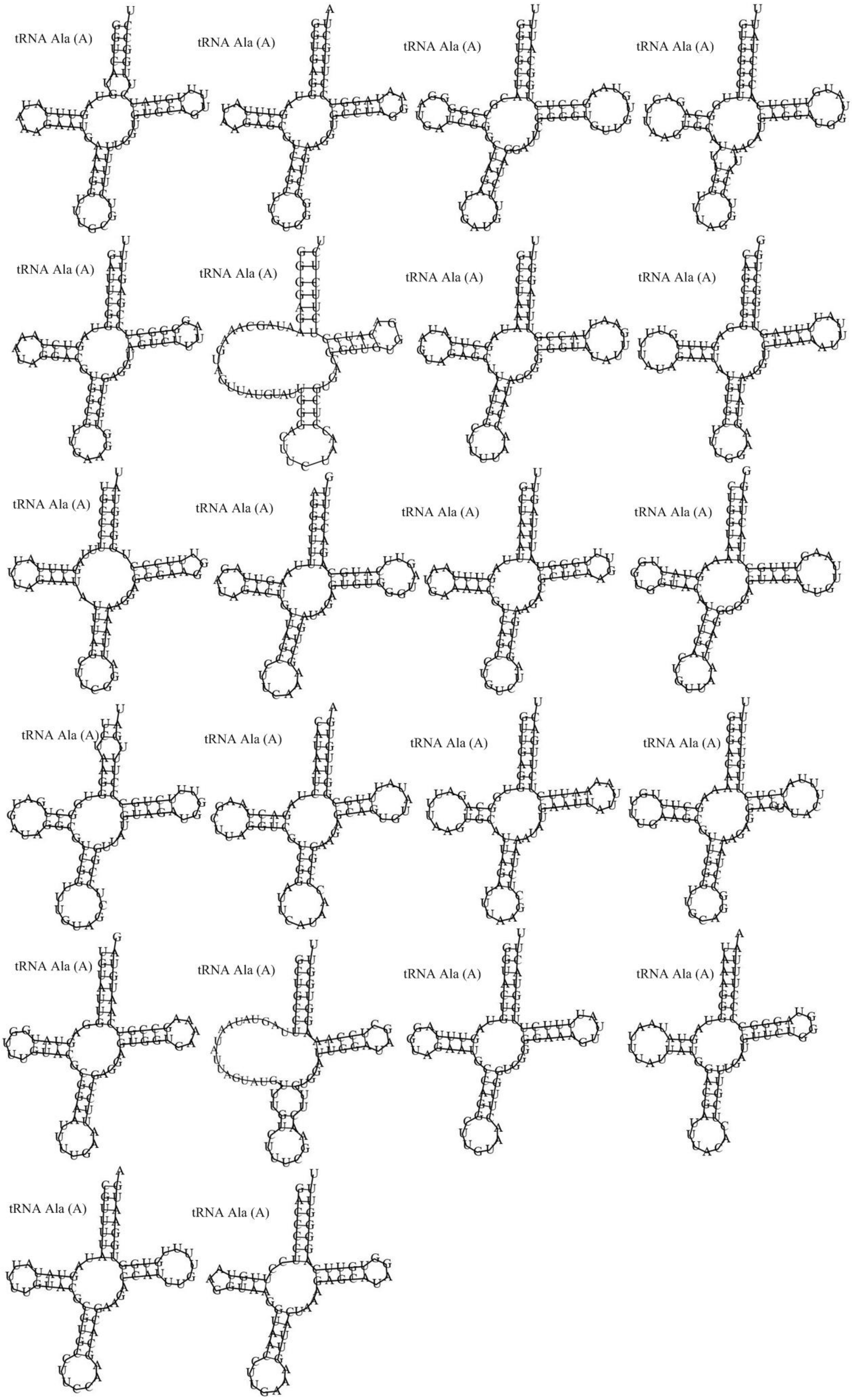

Supplementary Fig 2. Cloverleaf structures of the 23 mt tRNA genes deduced from the mitochondrial genome of *Potiarca pilula*.

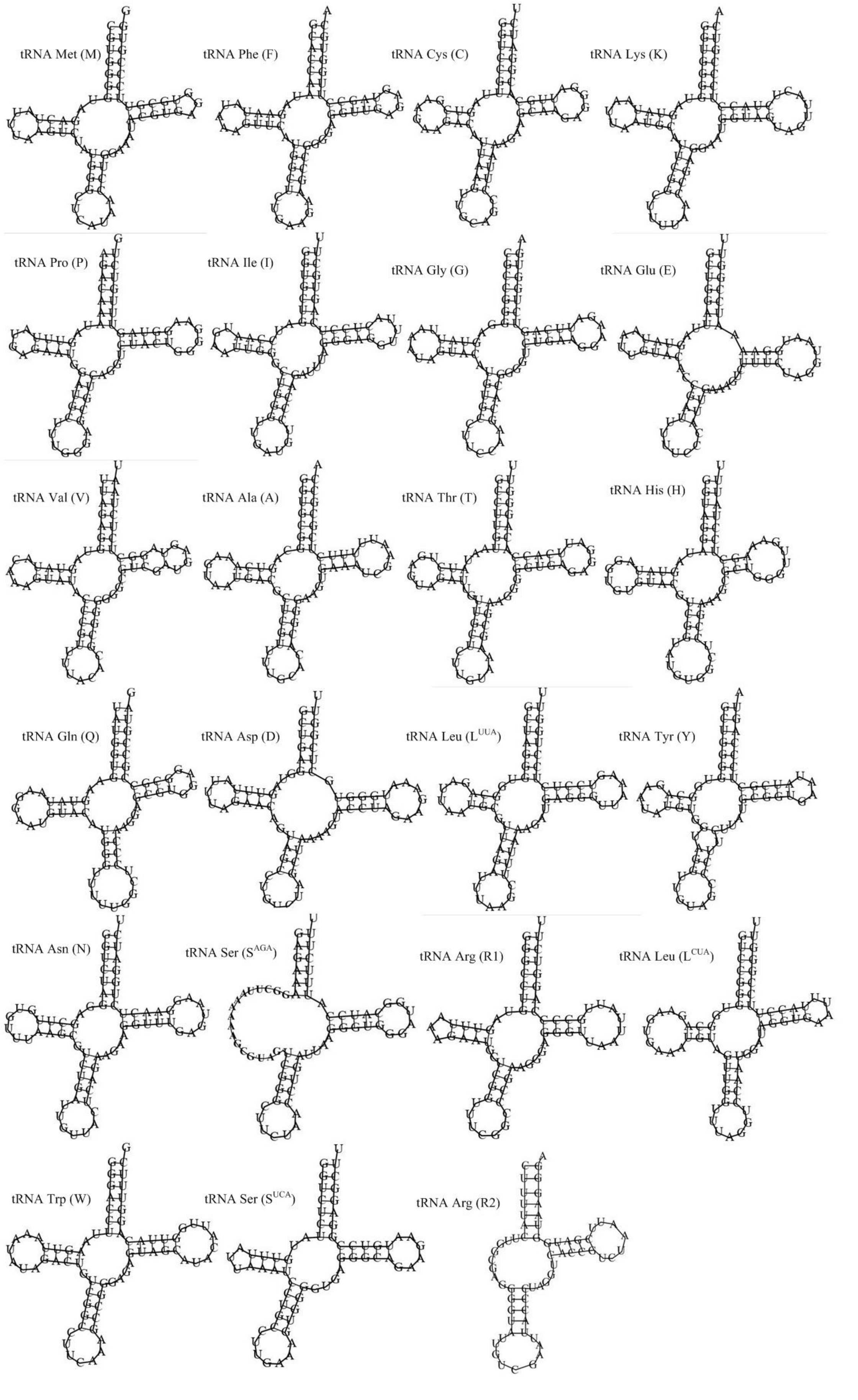

Supplementary Fig 3. Stem-loop structures of the tandem repeat motif (represented by the first repeat unit) in the non-coding region of *Trisidos kiyoni* and *Potiarca pilula* mt genomes. A) The stem-loop structures of the tandem repeat motif with 256 bp in *Trisidos kiyoni* mt genome. B) The stem-loop structures of the first repeat family with 283 bp in *Potiarca pilula* mt genome. C) The stem-loop structure of the second repeat family with 183 bp in *Potiarca pilula* mt genome.

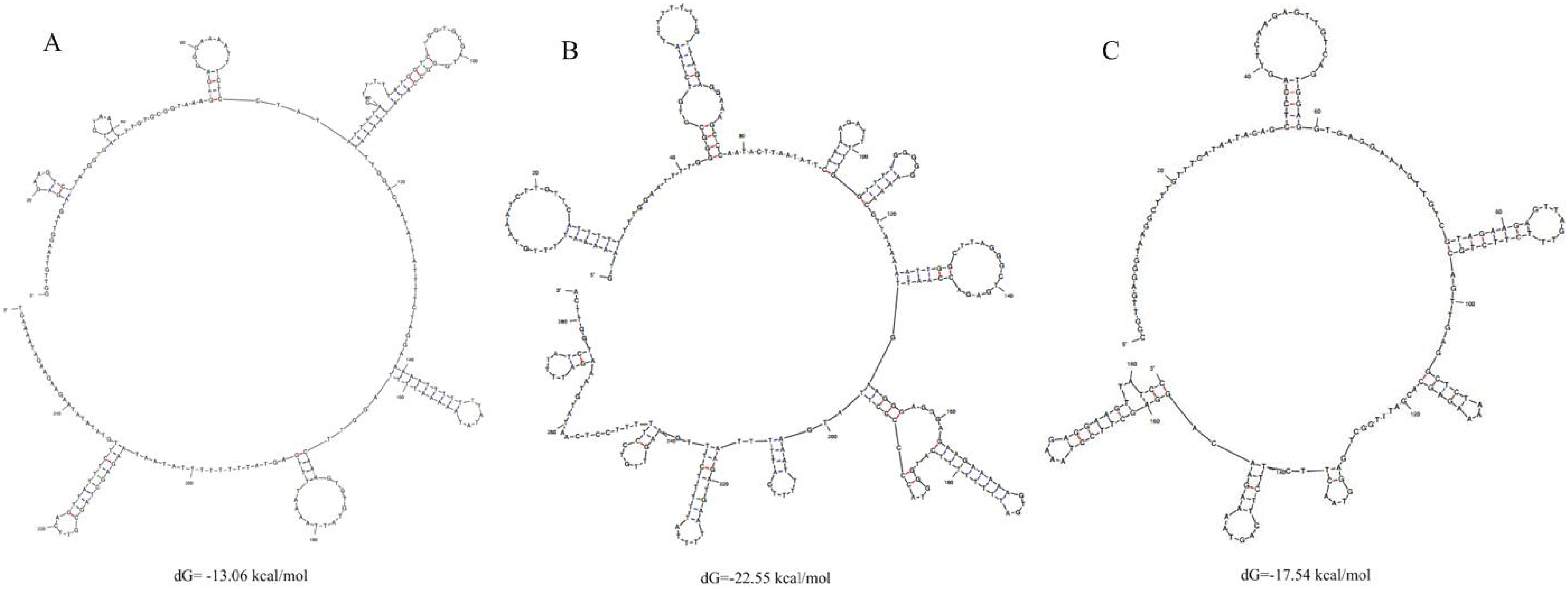

Supplement: Supplementary Information [file srep33794-s1.pdf]
